# The Buffalo Water Supply

**Published:** 1912-01

**Authors:** 


### The Buffalo Water Supply

IT is rather disconcerting, after the lavish expenditure of money and the pushing of the tunnel far beyond the old intake, to learn from Major Bissell's bacteriologic tests, that Buffalo has practically no better water than before. Not knowing exactly how much further expense will be required to rebuild the pumping station, pay damages for the men killed during its construction and bring the equipment to its completion, we can not speak with arithmetic accuracy but it is a fair estimate that every average man, woman and child in Buffalo will pay somewhere between five and ten dollars for an experiment which has failed.

The question is: What are we going to do about it? Short of the ideal solution of establishing sand filtration and disregarding other engineering possibilities which would virtually nullify the whole work up to the present, we admit our inability to answer the question. However, two suggestions may be made, with the understanding that they are purely tentative:

1. The large number of vessels passing in and out of the harbor, above the present intake is a serious danger, not so much on account of the discharges from their own crew and passengers, as of the foul water which they draw from the harbor, with only a few rods washing in fairly pure water. There are only four ports in the world which have more shipping than Buffalo. Either a new channel should be established below the intake or the intake should be moved a quarter of a mile farther south.

2. As much of the trouble with the water is from roiliness, it is worth considering whether a break wall, running at right angles into the lake from the present one might not furnish a fairly quiet pool, which could be dredged deeply at intervals. Such an arrangement ought to protect from gross contamination along the southern shore, while the natural current would protect from Canadian sewage.

